# Supplementary figures and images for: Cloning of the DlERF10 gene from Diospyros lotus L. and cold tolerance analysis of the DlERF10 gene in transgenic tobacco plants
Source: PLoS One. 2025 Mar 3;20(3):e0314135. doi: 10.1371/journal.pone.0314135 (PMC11875332; doi:10.1371/journal.pone.0314135)

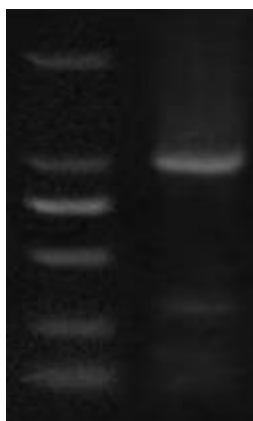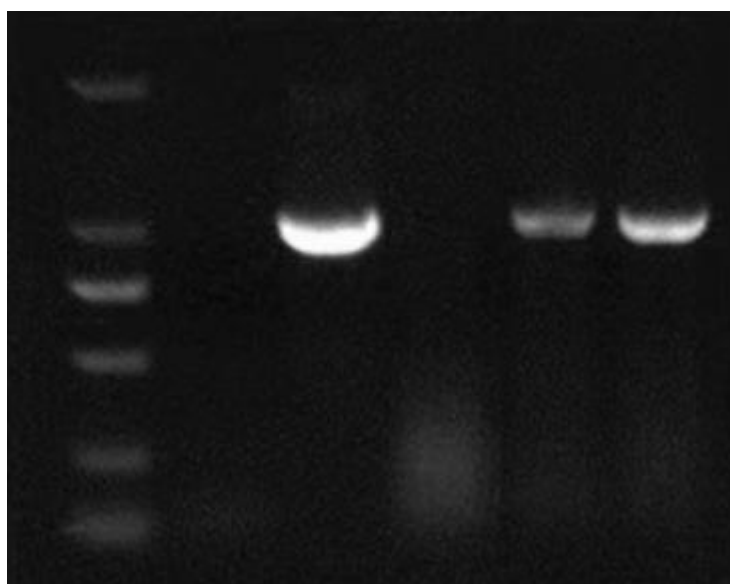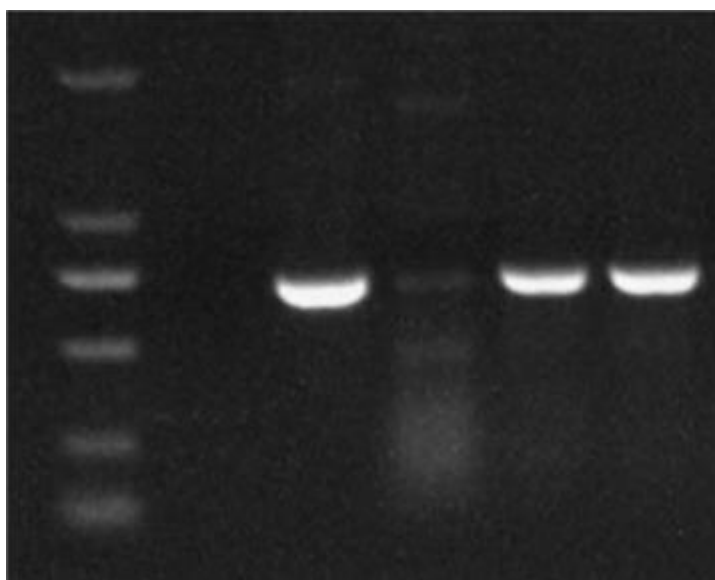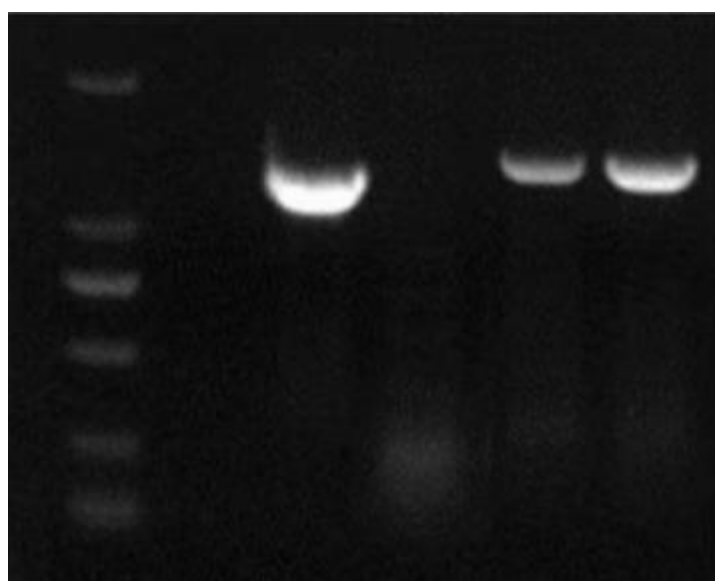

Supplement: S1 Fig — (PDF) [file pone.0314135.s001.pdf]
