# Supplementary material for: Cloning of the DlERF10 gene from Diospyros lotus L. and cold tolerance analysis of the DlERF10 gene in transgenic tobacco plants
Source: PLoS One. 2025 Mar 3;20(3):e0314135. doi: 10.1371/journal.pone.0314135 (PMC11875332; doi:10.1371/journal.pone.0314135)

بنام خدا

Cloning of the DlERF10 gene from Diospyros lotus L. and cold resistance analysis of the DlERF10 gene in transgenic tobacco plants

Line 95: there is no appropriate reference for text: Xu et al. 2021

Line 237-239: there is no figure legend for figure 1

Line 241-242: what is second conserve DLERF10 protein (the figure 2 legend is ambiguous)

Line 314: what is the word and its number: proteins[45].

Line 351: figure 11 - there are no determined what figure is A, B or C

Line 488: please write correct the word: *D. lous*.

Line 497: has been demonstrated in previous studies.......what is previous study?

Line 536: The Gao YD et al. (2020) is no searchable

Line 562: The Wei et al (2020) is no searchable

This reference not used in the text:

Ohme-Takagi M, Shinshi H. Ethylene-inducible DNA binding proteins that interact764 with an ethylene-responsive element. The Plant Cell, 1995, 7(2): 173-182. DOI:765 10.1105/tpc.7.2.173.766

The discussion odf manuscript is very tall and boring for the readership

Sincerely yours

Bahram Kazemi PhD, Professor at Shahid Beheshti University of Medical Sciences

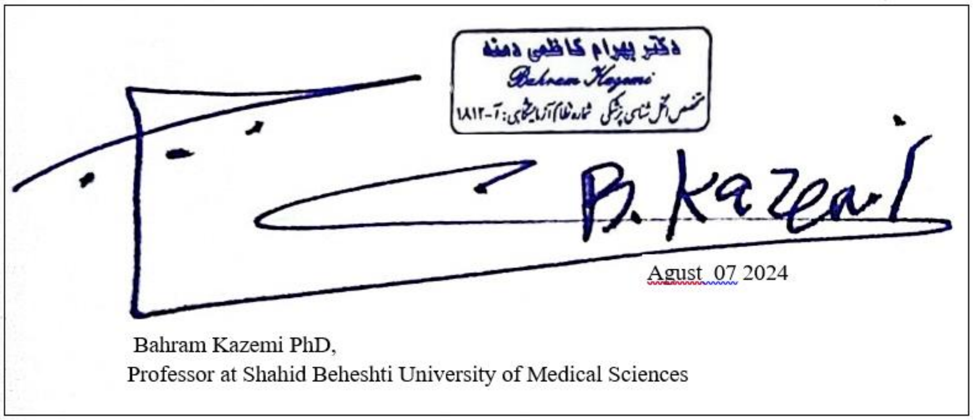

Supplement: Attachment — Submitted filename: Cloning of theDlERF10.pdf [file pone.0314135.s002.pdf]
